# Genetic diversity and drug resistance of HIV-1 among infected pregnant women newly diagnosed in Luanda, Angola

**DOI:** 10.1371/journal.pone.0225251

**Published:** 2019-11-26

**Authors:** Cruz S. Sebastião, Zoraima Neto, Carlos S. de Jesus, Marinela Mirandela, Domingos Jandondo, José C. Couto-Fernandez, Amilcar Tanuri, Joana Morais, Miguel Brito

**Affiliations:** 1 NOVA Medical School, Faculdade de Ciências Médicas, Universidade NOVA de Lisboa, Lisboa, Portugal; 2 Laboratório de Biologia Molecular, Instituto Nacional de Investigação em Saúde, Luanda, Angola; 3 Instituto Superior de Ciências da Saúde, Universidade Agostinho Neto, Luanda, Angola; 4 Centro de Investigação em Saúde de Angola, Luanda, Angola; 5 Laboratorio de AIDS & Imunologia Molecular, Instituto Oswaldo Cruz, Rio de Janeiro, Brazil; 6 Laboratório de Virologia Molecular, Departamento de Genética, Universidade Federal do Rio de Janeiro, Rio de Janeiro, Brasil; 7 Faculdade de Medicina, Universidade Agostinho Neto, Luanda, Angola; 8 Health and Technology Research Center, Escola Superior de Tecnologia da Saúde de Lisboa, Instituto Politécnico de Lisboa, Lisboa, Portugal; University of Cincinnati College of Medicine, UNITED STATES

## Abstract

Monitoring genetic diversity and drug resistance mutations (DRMs) is critical for understanding HIV epidemiology. Here, we report HIV-1 genetic diversity and DRMs in blood samples from 42 HIV-positive pregnant women naive to antiretroviral therapy (ART), in Luanda. The samples were subjected to nested-PCR, followed by sequencing of HIV-1 *pol* gene, targeting the protease and reverse transcriptase fragments. HIV-1 diversity was analyzed using the REGA HIV-1 subtyping tool and DRMs were identified using the Calibrated Population Resistance tool. A total of 34 sequences were obtained. The data revealed wide HIV-1 subtypes heterogeneity, with subtype C (38%, 13/34) the most frequent, followed by the subtypes F1 (18%, 6/34), A1 (9%, 3/34), G (9%, 3/34), D (6%, 2/34) and H (3%, 1/34). In addition, recombinants strains were detected, with CRF02_AG (6%, 2/34) the most frequent, followed by CRF37_cpx, F1/C, A1/G and H/G, all with 3% (1/34). A total of 6/34 (18%) of the sequences presented DRMs. The non-nucleoside reverse transcriptase inhibitors presented 15% (5/34) of resistance. Moreover, 1/34 (3%) sequence presented resistance against both non-nucleoside reverse transcriptase inhibitors and nucleoside reverse transcriptase inhibitors, simultaneously. Despite the small sample size, our results suggest the need to update currently used ART regimens. Surveillance of HIV-1 subtypes and DRMs are necessary to understand HIV epidemiology and to guide modification of ART guidelines in Angola.

## Introduction

The human immunodeficiency virus (HIV) has become a major global public health problem [1], affecting about 36.9 million people in the world [2]. In Angola, a total of 310,000 cases were reported in 2018 [2]. HIV is classified into types (HIV-1 and HIV-2), groups (M, N, O and P), subtypes (A-D, F-H, J and K), sub-subtypes (A1, A2, F1 and F2), circulating recombinant forms (CRFs) and unique recombinant forms (URFs) [3]. HIV-1 is responsible for the vast majority of HIV infections [4]. All subtypes of HIV-1 group M (except B), several CRFs and URFs have been described in Angola [5–11].

Universal access to antiretroviral therapy (ART) has successfully decreased mortality and morbidity associated with HIV [2,12]. The first-line of the ART drugs used in Angola includes the nucleoside reverse transcriptase inhibitors (NRTIs), tenofovir (TDF) and lamivudine (3TC), and a non-nucleoside reverse transcriptase inhibitor (NNRTI), either efavirenz (EFV) or nevirapine (NVP) [13,14]. In addition, zidovudine (AZT) has been used to prevent vertical transmission [13,14].

The emergence of HIV-1 subtypes with drug resistance mutations (DRMs) during pregnancy represents a challenge for the efficacy of ART, especially in low- and middle-income countries [15]. There is a lack of recent data on HIV-1 genetic diversity and prevalence of DRMs in Angola [15,16]. In this study, we investigated the genetic diversity and DRM prevalence in blood samples from HIV-positive pregnant women naive to ART in Luanda, to better understand HIV epidemiology and to allow a timely modification of ART guidelines in Angola.

## Materials and methods

### Study design and sample collection

A cross-sectional study was carried out at the Lucrecia Paim Maternity clinic, located in Luanda, capital city of Angola, during the months of April to June of 2018. The study involved 1612 pregnant women who were screened for HIV infection using the rapid antibody detection test Determine HIV1/2^™^ (Alere, Japan) and the Unigold^™^ HIV (Trinity Biotech, Ireland) during prenatal care. Sociodemographic characteristics and blood samples were collected from HIV-positive pregnant women. The main criterion for inclusion of HIV-positive pregnant women was that they had not been previously exposed to any ART. The blood samples were collected in a tube with EDTA, centrifuged and the plasma was aliquoted and stored at -80°C. The blood samples preparation was performed at the Molecular Biology Laboratory, of the National Institute for Health Research of Angola (INIS). Following the recommendations of the National Institute of Fighting against AIDS (INLS), the HIV-positive women, were prescribed ART with TDF, 3TC and EFV, and were medicated with AZT until child birth [13,14].

### RNA extraction, cDNA synthesis, PCR and sequencing

Total viral RNA was extracted from 140μL of plasma using QIAamp Viral RNA kit (QIAGEN, Germany) following the manufacturer instructions. The cDNA synthesis was carried out using 10μL of the RNA in a final reaction volume of 20μL. The mix contained 25mM DNTP mix, 5X M-MLV buffer, 10mM of dithiothreitol (DTT), 40U of RNase OUT^™^ (Life Technologies, USA), 0.1mM of MMRTR6 primer (5’-TTTTACATCATTAGTGTGGG-3’), and 200U of M-MLV enzyme (Life Technologies, USA) [17].

The obtained cDNA was subjected to a nested-PCR, targeting the protease (PR) and reverse transcriptase (RT) fragments of the HIV-1 *pol* gene, with an expected size of 1302 bp, using the protocol previously described [17]. Successful amplification was checked using a 1% agarose gel. The amplicons were purified using the NZYGelpure Kit (Nzytech, Portugal), and sequenced using the ABI BigDye Terminator v3.1 reaction kit (Applied Biosystems, USA). For each sample, eight primers were used for the complete sequencing of the PR (nucleotide range: 2253–2549) and the first 335 codons of RT (nucleotide range: 2550–3554), considering the genome of the *HXB2* strain (nucleotide range: 2252–3554) [17]. Sequencing was performed on an ABI 3500 sequencer (Applied Biosystems, USA) at the Molecular Biology Laboratory of the INIS, in Luanda.

### HIV-1 subtyping, phylogenetic and resistance mutation analysis

The electropherograms were analyzed using the software RECALL v2.25 [18]. Classification of HIV subtypes was conducted using the REGA HIV-1 subtyping tool v3.0 (http://dbpartners.stanford.edu:8080/RegaSubtyping/stanford-hiv/typingtool/) [19]. The nucleotide sequences obtained were aligned with HIV-1 M-group nucleotide sequences downloaded from NCBI (https://www.ncbi.nlm.nih.gov/nuccore/) and Los Alamos (https://www.hiv.lanl.gov/content/index) databases. The sequences obtained in the study were deposited in GenBank (NCBI) and were assigned the accession numbers MK543512 to MK543545. A phylogenetic tree was inferred using the Neighbor-Joining (NJ) method [20], with Tamura-Nei genetic distances [21]. Clade support was assessed using 1000 bootstrapped replicates [22]. The phylogeny was estimated using MEGA software v7.0 [23], and recombinant viruses were characterized by boot scanning using SimPlot [24].

Drug resistance mutations to HIV-1 were identified using the Calibrated Population Resistance tool (CPR) v8.0 (https://hivdb.stanford.edu/cpr/form/PRRT/) [25]. Additionally, analyses of drug resistance profile were performed using the Stanford genotypic resistance interpretation algorithm (https://hivdb.stanford.edu/hivseq/by-sequences/) [26].

### Statistical analysis

Chi-square (X^2^) tests were performed to evaluate the association between sociodemographic characteristics and HIV prevalence at 5% statistical significance in the SPSS v25 statistical program (IBM SPSS Statistics, USA).

### Ethical considerations

The pregnant women were informed of the study and consented (oral and written) to participation and follow-up until delivery. Consent from parents or guardians of the minors (under 15 years) was also obtained. All pregnant women underwent pre- and post-test counseling individually. The HIV study results were provided to the clinical staff to ensure appropriate patient clinical management. The study protocol was reviewed and approved by the National Ethics Committee of Angola (nr.13/2018), general directorate from Lucrecia Paim Maternity clinic (nr.083/GDG/MLP/2018) and the Ethics Research Committee of the NOVA Medical School/Faculdade de Ciências Médicas—Universidade NOVA de Lisboa (nr.51/2019/CEFCM).

## Results

### Sociodemographic characteristics

From the 1612 pregnant women tested for HIV, 42 (2.6%) tested positive and not been exposed to any ART previously. All pregnant women were from Luanda province. A total of 25/42 HIV-positive pregnant women were in the age group of 25–34 years, 18/42 had basic and secondary education, respectively, and 19/42 were unemployed. Moreover, a total of 12/42 pregnant women were in the second trimester of gestation, followed by 11/42 in the third trimester and 10/42 pregnant women diagnosed and the sample was obtained just before labor (Table 1).

**Table 1:** Sociodemographic characteristics of pregnant women tested for HIV in Luanda, Angola, 2018.

Characteristics	Pregnant tested	HIV prevalence		X^2^	P-Value
		Negative	Positive		
Age group (years)					
<15	5/1612	4 (80.0%)	1 (20.0%)	11.985	0.017^a^
15–24	610/1612	602 (98.7%)	8 (1.3%)		
25–34	750/1612	725 (96.7%)	25 (3.3%)		
35–44	246/1612	238 (96.7%)	8 (3.3%)		
>44	1/1612	1 (100%)	0 (0.0%)		
Residence (Municipality)					
Luanda	643/1612	623 (96.9%)	20 (3.1%)	7.049	0.424
Viana	408/1612	400 (98.0%)	8 (2.0%)		
Belas	188/1612	181 (96.3%)	7 (3.7%)		
Kilamba Kiaxi	222/1612	216 (97.3%)	6 (2.7%)		
Sambizanga	14/1612	13 (92.9%)	1 (7.1%)		
Cazenga	108/1612	108 (100.0%)	0 (0.0%)		
Cacuaco	26/1612	26 (100.0%)	0 (0.0%)		
Icoli Bengo	3/1612	3 (100.0%)	0 (0.0%)		
Level of education					
Illiterate	72/1612	72 (100.0%)	0 (0.0%)	2.650	0.449
Basic	616/1612	598 (97.1%)	18 (2.9%)		
Secundary	633/1612	615 (97.2%)	18 (2.8%)		
Higher	291/1612	285 (97.9%)	6 (2.1%)		
Occupation					
Unemployed	733/1612	714 (97.4%)	19 (2.6%)	1.124	0.570
Worker	478/1612	463 (96.9%)	15 (3.1%)		
Student	401/1612	393 (98.0%)	8 (2.0%)		
Gestational Age (trimester)					
First	102/1612	93 (91.2%)	9 (8.8%)	33.526	0.000^a^
Second	176/1612	164 (93.2%)	12 (6.8%)		
Third	645/1612	634 (98.3%)	11 (1.7%)		
Parturient^b^	689/1612	679 (98.5%)	10 (1.5%)		

^a^ Chi-square (X^2^) statistics are significant (P<0.05).

^b^ Pregnant woman tested for HIV just before labor.

### Genetic diversity analysis

**Fig 1:**
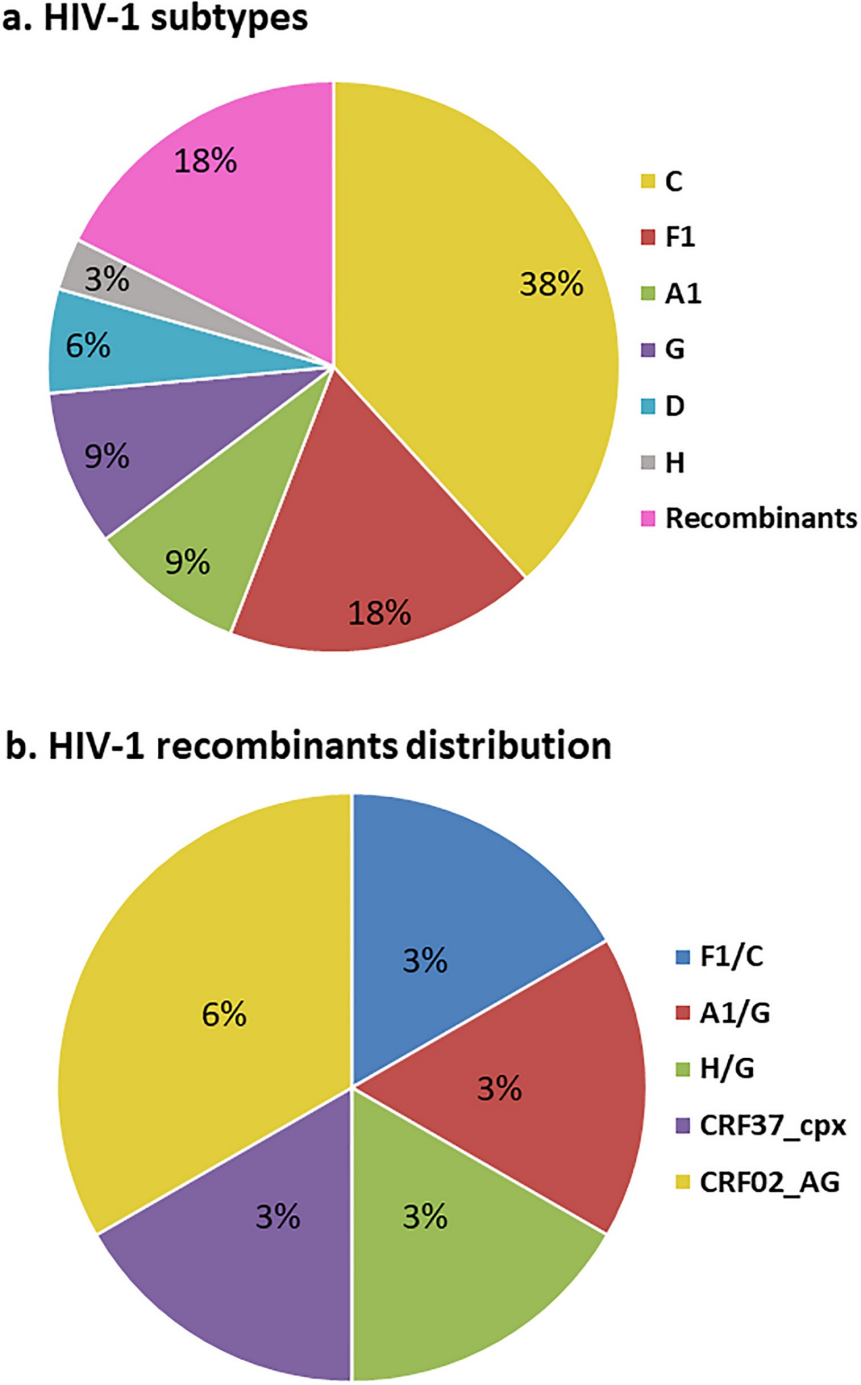
HIV-1 diversity among infected pregnant women in Luanda, Angola, 2018. The analysis involved 34 nucleotide sequences. (A) Frequency of HIV-1 subtypes and recombinants. (B) Recombinants distribution. The subtypes were identified by the REGA v3.0, and recombinants strains by SimPlot.

From the 42 plasma samples subjected to nested-PCR, a total of 34 amplicons and respective sequences were obtained. It was not possible to obtain amplicons from the remaining 8 samples, even after repeated PCR attempts using different primers. The genotyping analysis revealed that 28/34 sequences were HIV-1 pure subtypes and 6/34 recombinants strains. From the detected pure subtypes, subtype C (38%, 13/34) was the most frequent HIV-1 subtype, followed by the subtypes F1 (18%, 6/34), A1 (9%, 3/34), G (9%, 3/34), D (6%, 2/34) and H (3%, 1/34). From the recombinants strains detected, CRF02_AG (6%, 2/34), was marginally more frequent, followed by CRF37_cpx, F1/C, A1/G and H/G, all with 3% (1/34) (Fig 1).

**Fig 2:**
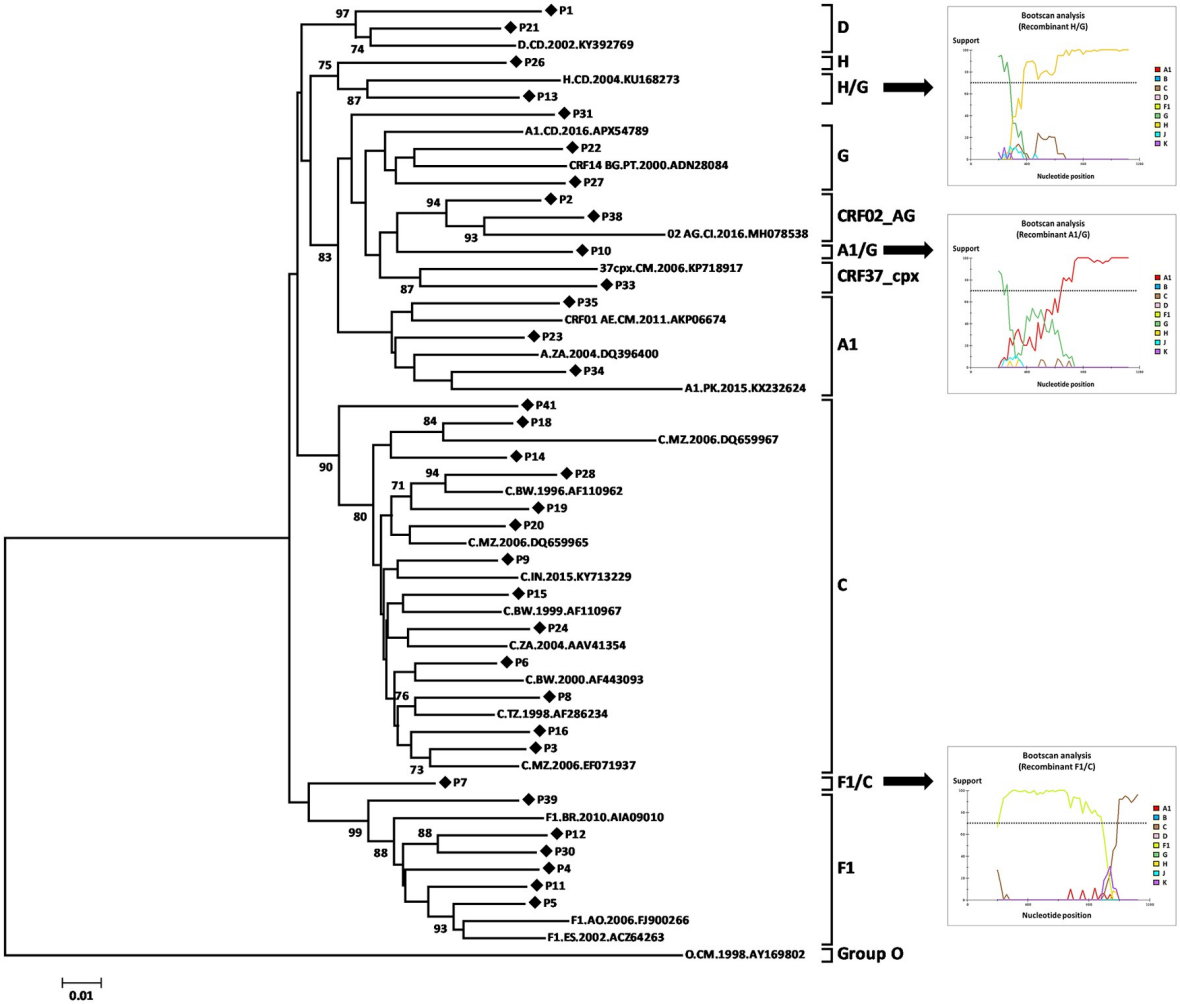
Evolutionary relationships of HIV-1 subtypes among infected pregnant women in Luanda, Angola, 2018. The analysis involved 56 nucleotide sequences. All HIV-1 strains identified in this study were from M-group. The phylogenetic tree was inferred using the NJ method, and Tamura-Nei genetic distances with 1000 bootstrap replicates. Analyses were conducted in MEGA v7.0. Bootstrap values more than 70% and recombinants strains are indicated.

Genetic distance analysis showed genetic similarity (more than 70%) of HIV-1 subtype C isolates with isolated from Botswana, Mozambique, Tanzania, South Africa and India. The subtype F1 was more similar to isolates from Brazil and Spain. The subtype A1 was most similar to isolates from Cameroon, South Africa, and Pakistan. The subtype G was most similar to isolates from Portugal, and subtype D, subtype H, and recombinant strain H/G to isolates from Democratic Republic of Congo. The recombinants strains A1/G, CRF02_AG, and CRF37_cpx were similar to isolates from Cameroon and Ivory Coast (Fig 2).

### Antiretroviral resistance mutations and resistance profile

The sequence analysis showed that 6/34 (18%) of the sequences presented DRMs. Of these, 5/34 (15%) sequences presented resistance to specific NNRTIs. Moreover, 1/34 (3%) sequence presented resistance against both NNRTIs and NRTIs, simultaneously. Mutations at positions K103N (2/34), G190A (2/34), Y181I (1/34), and P225H (1/34) were observed against the class of NNRTIs, whereas mutations at positions M41L, D67N, T69D and T215S were observed against NRTIs. A non-polymorphic protease inhibitor (PI) selected mutation was observed at position I85V. High levels of drug resistance were observed for NVP (5/34) and EFV (2/34), intermediate resistance for EFV (3/34) and AZT (1/34) and low resistance for Abacavir (ABC) (1/34) and TDF (1/34). In addition, the susceptibility profile associated to the second generation of NNRTIs was also identified and 1/34 and 3/34 sequence presented high- and low-resistance to Etravirine (ETR) and Rilpivirine (RPV), respectively (Table 2).

**Table 2:** Drug resistance mutations to NRTI, NNRTI and PI according to the HIV-1 subtypes and resistance profile.

Isolate	HIV-1 subtypes	Drug resistant mutations			Drug resistance profile		
		NRTI	NNRTI	PI	Low	Intermediate	High
P10	A1/G	-	G190A	-	ETR, RPV	EFV	NVP
P12	F1	-	K103N, P225H	-	ETR, RPV	-	EFV, NVP
P26	H	-	G190A	-	ETR, RPV	EFV	NVP
P27	G	M41L, D67N, T69D, T215S	Y181I	-	ABC, TDF	AZT, EFV	ETR, RPV, NVP
P31	G	-	-	I85V	-	-	-
P41	C	-	K103N	-	-	-	EFV, NVP

Antiretroviral drugs: AZT—Zidovudine; EFV—Efavirenz; ETR—Etravirine; NVP—Nevirapine; RPV—Rilpivirine; ABC—Abacavir; TDF—Tenofovir.

## Discussion

This study presents an important update on molecular epidemiology of circulating HIV-1 strains in Luanda. The HIV prevalence was 2.6% (42/1612). A significant difference in HIV positivity was observed between pregnant women of different age groups and pregnancy stage (P<0.05). On the other hand, residence, level of education and occupation did not show significant differences (P>0.05) (Table 1).

Genetic analysis of 34 isolates revealed a wide diversity of HIV-1 strains (Fig 1), similar to that observed in previous studies performed in Angola [5–11]. Though it is hard to prove statistical significance with our small sample size, our study indicates an increase in HIV-1 subtype C, but a slight decrease in subtype F1 in Luanda [5,8]. The genetic similarity of HIV-1 subtype C with isolates from Botswana, Mozambique, Tanzania, South Africa, India and the subtype F1 with isolates from Brazil and Spain (Fig 2), may be attributed to high mobility between countries or to the fact that, after colonial war, and the end of civil war in 2002, thousands of refugees returned to Angola [27].

The RT inhibitors are important components of ART regimen [12–14]. The identification of pre-treatment drug resistance in pregnant women naïve to ART (Table 2), may threaten ART based strategy to HIV control in Luanda [12–14]. The K103N and G190A mutations are associated with EFV and NVP resistance [28]. The Y181I and P225H mutations are often associated with second generation RPV and ETR resistance [29]. The thymidine analogue associated mutations (TAMs) at positions M41L and D67N have the greatest impact on susceptibility of AZT and Stavudine (d4T) [28]. The T69D mutation when present with T215S mutation, is associated with broad resistance to NRTIs [28]. The non-polymorphic PI-selected mutation at position I85V has minimal effects on PIs susceptibility [30,31].

The identification of K103N and G190A mutations, may be attributed to the long use of NNRTIs as part of the first-line ART regimens in Angola [13,14]. Displacement of people to countries where ART is available for a longer period also may help to explain the origin of the HIV-1 subtypes with DRMs in Luanda [27,32].

The reasons for PCR failure of 8/42 samples were not identified. It may be that the high HIV-1 genetic diversity compromised the binding of primers, even though they were targeted at highly conserved regions of the HIV-1. Other studies with more representative sampling monitoring of the HIV-1 subtypes and DRMs are necessary to guide a timely modification of ART guidelines in Angola. Despite the small sample size, our findings suggest that the Angolan Ministry of Health should prompt consideration of moving to generic integrase strand transfer inhibitors (INSTIs) in the ART regimen in Angola [33].

## Conclusions

Our results show a wide HIV-1 subtypes heterogeneity, with subtype C the most frequent. A total of 6/34 (18%) of the sequences presented DRMs. Of these, 15% (5/34) were associated with resistance against NNRTIs. Moreover, 1/34 (3%) sequence presented resistance against both NNRTIs and NRTIs, simultaneously. Our findings suggest the need to update currently used ART regimens. Better understanding is needed of emergence of HIV-1 subtypes and DRMs, to allow a timely modification of ART guidelines in Angola.
